# Parsimony of Publishers

**Published:** 1852-11

**Authors:** 


*Parsimony of Publishers.* — The following article, with the above caption, is from the N. Y. Med. Gazette. In common with the Med. Examiner, we received an order for Bernard & Huette's work on operative surgery, which, the writer of the order was at pains to designate, was to be *plain.* We immediately returned the order, declining to receive it.

We do not entirely concur with the editor of the Gazette in the concluding paragraph of his article. The *American* publishers of the London Lancet, belong in the same category:

"The last number of the *Medical Examiner* contains a 'palpable hit' at the publisher of the two numbers which have appeared of Bernard and Huette's Manual of Operative Surgery. The reviewer complains that the copies sent to the editors of the medical journals are 'in mourning,' being without the 'colors' to the plates, which they are expected to introduce to their readers. He facetiously remarks that he 'confesses to some weakness for the

*feathers* along with the *fuss*, and expects to see a book in the best dress that belongs to it.'

" A neighboring contemporary of ours called on this same publisher to examine his stock of foreign books, and was shown a copy of the first number of the work in question, with colored plates. While looking at it the publisher solicited a good notice of it in the forthcoming number of his quarterly, at the same time apologizing for not presenting a copy for the purpose, by assuring him that this usual courtesy had to be withheld, as he could not afford it! The uncolored copy sent to our friend, at Philadelphia, was therefore a stretch of liberality, for which he should be truly grateful.

" Such littleness merits reprobation for its bad policy, and is never betrayed by any *American* publisher. They are wiser in their generation, and hence never begin a work without being able to finish it."

